# Real-time procedure information sharing as a means to reduce perioperative anxiety in families of children undergoing elective surgery - a randomized controlled study

**DOI:** 10.1186/s12871-024-02581-y

**Published:** 2024-06-05

**Authors:** Linjun Yun, Yuanhong Li, Lu Yin

**Affiliations:** 1 Department of Anesthesiology, West China Hospital, West China School of Nursing, Sichuan University, Chengdu, 610041 Sichuan Province People’s Republic of China; 2 West China Tianfu Hospital, Sichuan University, Chengdu, 610041 Sichuan Province People’s Republic of China

**Keywords:** Real-time surgical process, Share, Family, Family anxiety, Satisfaction

## Abstract

**Objective:**

To investigate whether the surgical process information sharing system could alleviate the parental anxiety during a pediatric selective operation.

**Design:**

Randomized controlled trial.

**Methods:**

A questionnaire survey was conducted one day before surgery for the enrolled participants. Family members assigned to the intervention group received real-time process information sharing through service reminders during the surgical period, while the control group received standard perioperative education. The Pittsburgh Sleep Quality Index (PSQI) was used to evaluate sleep quality during the perioperative period, and the State of Cohesion-13 Scale (SOC-13) and Self-rating Anxiety Scale (SAS) were used to assess anxiety levels. Satisfaction levels during the perioperative period were assessed through a follow-up survey conducted one day after surgery.

**Results:**

The intervention group showed better scores in terms of PSQI, SOC-13, SAS, and postoperative satisfaction levels at various time points compared to the control group, with statistically significant differences observed (*P* < 0.05).

**Conclusion:**

Real-time process information sharing is effective in reducing perioperative sleep disorders and anxiety among family members of pediatric patients, as well as improving satisfaction levels. This approach not only establishes a process and mechanism for effective doctor-patient communication but also helps implement continuous perioperative care, thereby optimizing internet healthcare services.

## Background

Pediatric surgery is an important method of treating surgical diseases in children under 12 years of age. Compared to adults, pediatric surgeries carry higher medical risks, and both surgical and anesthesia procedures can cause significant psychological and physiological changes in children. Additionally, these procedures can also impose substantial mental and financial stress on family members. While healthcare professionals typically provide comprehensive care for patients, they often overlook the psychological condition and support needs of the family members [1]. Regardless of the size of the surgery, general anesthesia procedures are a significant source of stress for the families involved. As important sources of social support for the surgical patient, the mental well-being and resilience of family members play a crucial role in the patient’s physical and psychological recovery during the perioperative period [2]. Surgery is not only a major physical and psychological trauma for the patient but also represents a significant negative life event for family members [3].

The family is the most important source of social support for a child, and during the perioperative period, they have to cope with various uncertainties related to the surgery, and their expectations of seeing the child increase their anxiety levels [4]. Regardless of the type of surgery, the desire and expectation for real-time information about the surgery are particularly important to family members caring for the child. Mishandling of this information can cause extreme psychological distress and often leads to psychological stress reactions, with anxiety being a common manifestation. The psychological changes in one family member can affect other family members, and interventions targeting the entire family can indirectly benefit the patient [5]. Kim et al. [6] conducted a study on the correlation between preoperative anxiety and sedation requirements in 455 family members of children aged 0–6 years and found that the younger the child was, the higher the anxiety level of the family member. The isolated and closed environment outside the operating room can create a sense of helplessness and anxiety among family members. Previous research showed that over 97.6% of family members had a strong desire for information about the patient’s surgical process, and 40% of family members believed that obtaining surgical information could reduce anxiety. In the survey, family members reported increased nervousness and anxiety, especially before the surgery and on the night of the surgery, as well as sleep disturbances, increased heart rate, and unexplained feelings of fear [4].

The “Guiding Opinions of the State Council General Office on Establishing the Modern Hospital Management System“ [7] and the “National Nursing Career Development Plan (2016–2020)” issued by the former National Health and Family Planning Commission [8] both emphasize the need to strengthen the standardization and standard construction of hospital information systems, promote nursing informatization, improve medical service management, enhance medical services, optimize the medical process and patient experience, and actively explore innovative optimization of nursing processes and service forms. Modern nursing advocates a “health-centered” service concept, where the recipients of nursing services are not only patients but also individuals, families, communities, and society as a whole. In clinical nursing, healthcare professionals not only provide psychological care to patients but also pay attention to the psychological well-being of family members.

According to family systems theory, family members form an interactive system in which they mutually influence each other. The behaviors of one member can affect the cognitive and emotional changes of other members. The operating room is characterized by high personnel mobility and close contact among staff members, making it a weak link in hospital infection prevention and control efforts [9, 10]. How to avoid nosocomial infections while meeting the information needs of family members has become an important issue of widespread concern among surgical healthcare professionals. Therefore, it is imperative to optimize the surgical process, employ network information release of surgical procedures, meet social demands, and enhance the delivery of quality nursing services.

### Purpose

The purpose of this study is to investigate whether the surgical process information sharing system could alleviate the parental anxiety during a pediatric selective operation.

## Methods

### Ethical declaration

This study adhered to the provisions of the World Medical Association’s Declaration of Helsinki and the National Health and Family Planning Commission of the People’s Republic of China’s “Regulations on Ethical Review of Biomedical Research Involving Humans” during the research process. It has been approved by the Ethics Committee of West China Hospital of Sichuan University (approval number: 2022 − 389), April 29, 2022), and has been registered in the China Clinical Trial Center (registration number: ChiCTR2300074573, October 8, 2023), (http://www.chictr.org.cn)” May 4, 2022, the first group of patients was enrolled, and on August 10, 2023, the trial was retrospectively registered.” All subjects and their relatives were informed about this study and signed an informed consent form before surgery.

### Study design and sample

This study is a randomized controlled trial. Prior to this, we conducted a survey to determine the needs and opinions of the families of children undergoing surgery. The study will be conducted from May 2022 to October 2022 in the pediatric surgical ward of West China Hospital, Sichuan University, China. The participants’ general information will be randomly assigned by the researchers into two groups.

We did a pre-study survey, and it was conducted to assess parents’ demands regarding information sharing system and also to calculate sample size for the study. Based on the results of the pre-study survey, the current demand rate for information sharing during the perioperative period is approximately 79%. Assuming this demand rate will be achieved in this study, with a permissible error (Δ) of 5%, the estimated sample size is calculated as follows:

$$N={\left(\frac{U\alpha }{\varDelta }\right)}^{2}\times P\left(1-P\right)$$

The total sample size was 92, with 46 in the intervention group and 46 in the control group.

Inclusion criteria include children aged 0–12 undergoing elective surgery, voluntary participation in the study and acceptance of randomization, anticipated surgery time < 2 h, postoperative transfer to the Post Anesthesia Care Unit (PACU), and families of children with at least a primary school education level. Exclusion criteria included patients with a history of prior surgery, postoperative transfer to the intensive care unit (ICU) for further treatment, patients and their families with a history of psychiatric illness, the inability of the patient’s family to correctly use communication devices for receiving real-time process updates, the inability of the patient’s family to cooperate or having language barriers, and a history of psychiatric illness or hypertension in the child or their family.

### Randomization

The randomization in this study was to use a computer-generated random number. 50% of the numbers were odd numbers and the rest were even numbers. Then they were sorted by numerical order. The odd numbers were matched with control group, and the even numbers were matched with intervention group. There were 92 patients been examined, and finally, 3 of control group were and 2 of intervention group were excluded.

### Data collection methods

Data collection was conducted using general information forms, the PSQI, the SOC-13, the SAS anxiety scale, and satisfaction ratings. The reliability and validity of these forms (Chinese Ver.) were proved to be available [11–13]. Prior to the surgery, training on the use of the push notification system was provided to the family members. Group testing and detailed observation records were carried out by the researchers one day before the surgery.

### General information form

Researchers created a basic information form consisting of eight questions tailored to capture relevant data such as the child’s age, gender, duration of surgery, age of the family member, relationship with the child, education level, annual income, and marital status.

### Pittsburgh sleep quality index (PSQI)

PSQI was developed by Dr. Buysse, a psychiatrist at the University of Pittsburgh, and others in 1989 [14]. Liu Zhixi analyzed this scale [15], and the overall Cronbach’s α coefficient for the Chinese population was 0.87. The scale consists of 19 self-assessment items and 5 other scoring items, of which 18 self-assessment items are scored in 7 dimensions: sleep time, sleep duration, sleep efficiency, sleep disorders, and subjective sleep quality. Each dimension is scored from 0 to 3, and the cumulative score of each dimension is the total PSQI score (0 ∼ 21 points). The higher the total PSQI score is, the worse the sleep quality [16].

### State of cohesion-13 scale (SOC-13)

It is extracted from Antonovsky’s 29-item Sense of Coherence Scale [17]. The scale has 13 items, with a scoring standard of 1 ∼ 7 points, where 1 point represents “strongly disagree” and 7 points represent “strongly agree”. The total score of the scale is the sum of the raw scores of each item. The final score range is 13–91 points. A higher score indicates that the subject perceives a stronger sense of psychological coherence, and a lower score indicates the opposite. Bao Leiping’s analysis results show that the Cronbach’s α value of the SOC-13 scale in China is 0.76 [18].

### Self-rating anxiety scale (SAS) [16]

SAS was developed by William W.K. Zung. It is a standard for anxiety assessment used to measure the severity of anxiety and its changes during treatment. The SAS contains 20 items, with the main statistical indicator being the total score. The raw score is obtained by adding the scores of the 20 items; the standard score is obtained by multiplying the raw score by 1.25 and rounding to the nearest whole number. According to the Chinese norm results, the cutoff value for the SAS standard score is 50 points, with 50 ∼ 59 points indicating mild anxiety, 60 ∼ 69 points indicating moderate anxiety, and 70 points and above indicating severe anxiety. The Cronbach’s α coefficient of the SAS scale in China is 0.777 [19].

### Satisfaction score

This score includes a question about the satisfaction of the patient’s family with the text message reminder received after the surgery. “0” is defined as “completely dissatisfied”, and “100” is defined as “very satisfied”.

### Unawareness

The relevant researchers need to understand the grouping and data situation of all patients and send part of the research data to the families of the patients. Due to the nature of the research intervention, the families of the patients and the researchers cannot ignore the intervention (information sharing) because they need to participate openly.

### Data collection

Eighty-seven patients who met the inclusion criteria were prospectively identified. The researchers provided information, purpose, and significance about the study. On the day of surgery, basic data were collected using a general information form. The SAS scale was used to assess the anxiety of the families of the patients at six time points as follows: T0: one day before surgery; T1: when the patient was taken from the ward to the waiting area for patients, a nurse would scan the code on wristband and this time can be called as “waiting outside the operating room”; T2: as soon as the patient was taken into the operating room, the anesthetist will click the “entering room” option in the computer, and this time can be called as “entering the operating room”; T3: when the first time of surgeons cutting the skin, the anesthetist will click the “surgery start” option in the computer, and this time can be called as “the start of surgery”; T4: as soon as the end of suturing the skin the anesthetist will click the “surgery end” option in the computer, and this time can be called as “the end of surgery”; T5: when patient stayed in the PACU for at least 30 min, the nurse who was responsible for this patient would assess him/her if he/she could return to the ward, then the nurse will click the “leave the PACU” option in the computer if the assessment was passed, and this time can be called as “returning to the ward”. All of the information were exported from anesthesia system. The parents only received the time points above mentioned and the details on surgical steps will not be provided to them.

The SOC-13 score was used to assess the psychological state at T2, T3, and T5. The PSQI scale was used to assess the sleep quality of the families of the patients one day before surgery and the night of the day after surgery. The next morning, an anesthetist who was unaware of the grouping situation scored the patients would make the assessment. The satisfaction score was used to revisit perioperative satisfaction at the bedside one day after surgery.

### Control group

The control group routinely underwent pre-anesthetic nursing visits and perioperative-related education (approximate surgical steps, required time, waiting location, etc.) one day before surgery. During the surgery, they relied on regular communication with doctors and nurses.

### Experimental group

Unlike the control group, the experimental group shared real-time surgical progress information via a mobile application during the surgery. The specific plan is as follows: communicate with the families of the patients again one day before surgery and explain the specific process and risks of the surgery, agreeing to participate and share progress during the surgery. Ensure that the families understand the purpose of the surgery, communicate with them about each stage of the surgery, explain the details of the surgery, provide important information about the use of drugs and equipment, and answer any questions they may have. Information sharing channels (QQ, WeChat, email) were established, and the real-time status of the patient’s surgery collected from the surgical anesthesia system at five time points (T1, T2, T3, T4, and T5) was uploaded to the platform for integration and push to the families’ mobile phones. At the same time, related perioperative health education was sent. The families of the push group can obtain real-time updates on the progress of the child’s surgery through the information-sharing channel.

### Primary and secondary research endpoints

The main purpose of this study is to analyze in detail the degree of attention of the families to the surgical process of the patients during the perioperative period to provide a basis for future medical staff to manage the surgical process of the patients during the perioperative period and to formulate targeted measures. The perioperative experience of the patients and their families should be fully improved.

The secondary goal of this study is to alleviate the psychological burden of the families of the patients during the waiting period of the perioperative period, improve the hospital environment, and increase patient satisfaction.

### Statistical analysis

Data analysis was performed using the Statistical Package for Social Sciences (SPSS) version 22.0. Categorical variables were represented by absolute frequency and relative frequency, and continuous variables were represented by mean and standard deviation, according to normal distribution or nonnormal distribution. The student’s t test was used to compare the level of anxiety variables (numerical variables) and the remaining quantitative variables between groups. The chi-square test was used to test the correlation between qualitative variables and group variables. When the result (anxiety) was considered an ordered qualitative variable, the Mann‒Whitney test was used to compare the results (anxiety) of the two groups. A significance level of 5% was considered.

## Results

**Fig. 1:**
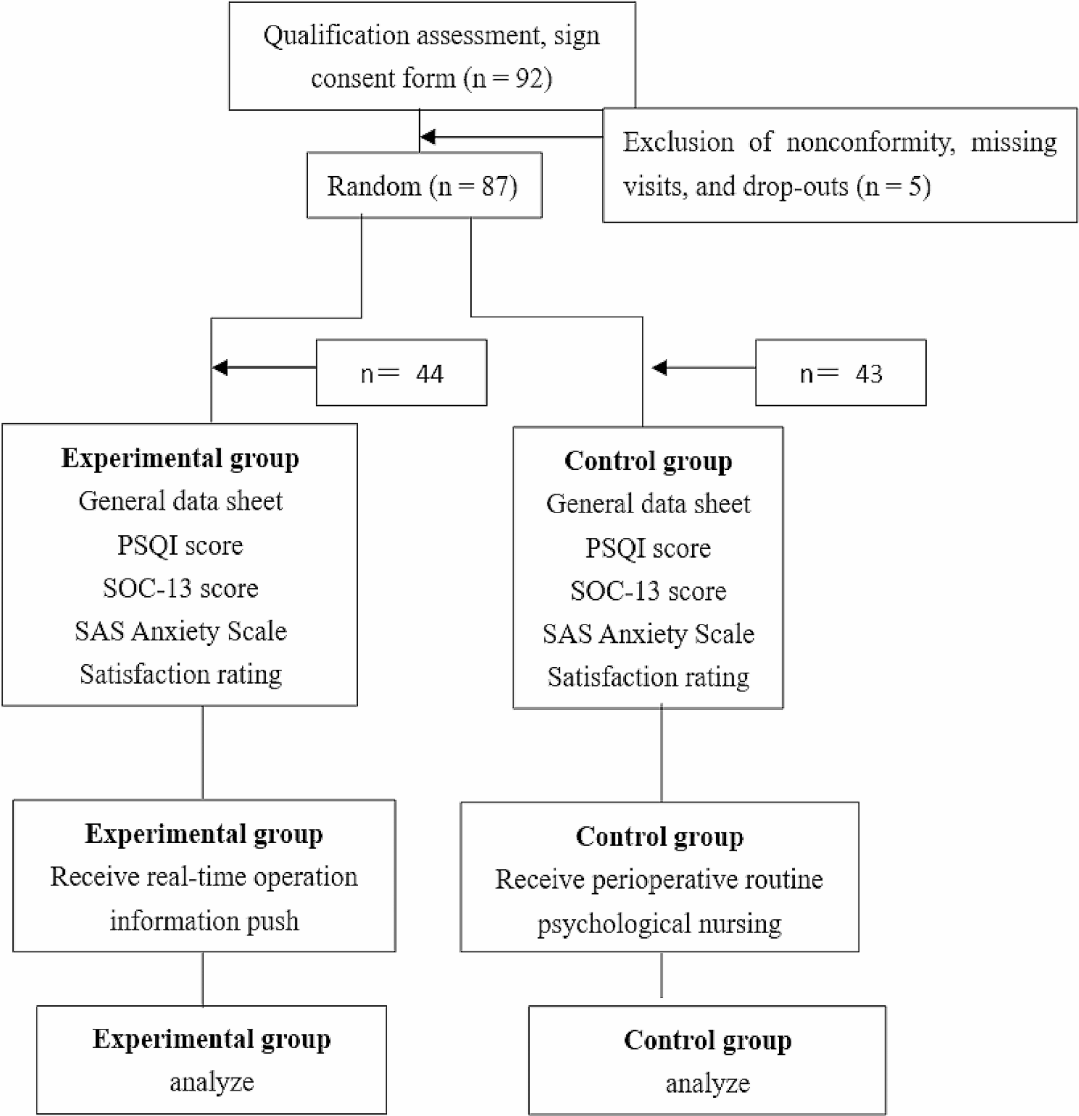
Flowchart of the study summarizing the steps used for patient selection

A total of 92 cases were included in this study. 5 cases were excluded (2 cases with surgery times exceeding 2 h; 2 cases withdrew midway; 1 case was lost to follow-up), resulting in a final total of 87 cases(see Fig. 1). The sociodemographic characteristics and surgery times were similar between the push group and the control group (*P* > 0.05; Table 1/Table 2).

**Table 1:** Sociodemographic characteristics of the children and their families

Items	Control group (*N* = 43)	Experimental group (*N* = 44)	t/χ^2^	*P*
**Age, year**	4.47 ± 3.225	4.41 ± 3.252	-0.081	0.936
**Sex**			-0.01	0.921
Male	23(53.5%)	24(54.5%)		
Female	20(46.5%)	20(45.5%)		
**Age of representatives, year**	39.47 ± 13.821	38.14 ± 13.086	-0.461	0.646
**Relationship with children**			1.674	0.643
Parents	27(62.8%)	28(63.6%)		
Not the same generation	11(25.6%)	11(25%)		
The same generation	3(7.0%)	1(2.3%)		
Employment relationship	2(4.6%)	4(9.1%)		
**Education of representatives**			6.373	0.272
Primary school	7(16.3%)	7(15.9%)		
Junior high school	8(18.5%)	12(27.2%)		
High school	10(23.3%)	6(13.6%)		
Junior College degree	10(23.3%)	5(11.4%)		
Bachelor degree	7(16.3%)	9(20.5%)		
Master degree or higher	1(2.3%)	5(11.4%)		
**Surgical type**			9.222	0.156
Strabotomy	11(25.6%)	15(34.1%)		
Tonsillectomy	6(14.0%)	5(11.4%)		
Phalloplasty	3(7.0%)	10(22.7)		
Laparoscopic descending fixation of cryptorchidis	6(14.0%)	6(13.6)		
Tylectomy	6(14.0%)	1(2.3%)		
Herniorrhaphy	5(11.6%)	2(4.5)		
Pyeloureteroplasty	6(14.0%)	5(11.4)		
**Annual income, RMB**			3.976	0.264
<50,000	9(20.9%)	7(15.9%)		
50,000-100,000	11(25.6%)	11(25%)		
100,000-150,000	19(44.2%)	15(34.1%)		
>150,000	4(9.3%)	11(25%)		
**Marital status**			1.738	0.419
Married	34(79.1%)	36(81.8%)		
Divorced	8(18.6%)	5(11.4%)		
Verwitwet	1(2.3%)	3(6.8%)		

N: number of patients; SD: standard deviation. * Student’s t test. Mann‒Whitney U test. Kruskal‒Wallis H test

**Table 2:** Comparison of surgical times between the two groups of children

Items	Control group(*N* = 43)	Experimental group(*N* = 44)	t	*P*
Operation time	40.84 ± 28.99	34 ± 26.87	-1.141	0.257

### Comparison of PSQI scores between the two groups

The PSQI scores of the push group patients were lower than those of the control group both one day before surgery and on the night after surgery (*P* < 0.05), as shown in Table 3.

**Table 3:** Comparison of PSQI sleep quality scores of the families during the perioperative period

Items	Control group(*N* = 43)	Experimental group(*N* = 44)	t	*P*
PSQI score of 1 day before surgery	12.58 ± 3.18	10.14 ± 4.07	-3.119	0.002
PSQI score on postoperative night	11.98 ± 4.55	8.50 ± 2.96	-4.235	<0.001

### Comparison of SOC-13 scores between the two groups

The scores of the intervention group at T2, T3, and T5 were significantly lower than those of the control group (*P* < 0.05), as shown in Table 4.

**Table 4:** Comparison of SOC-13 scores of the families during the perioperative period

Items	Control group(*N* = 43)	Experimental group(*N* = 44)	t	*P*
T2	31.56 ± 16.65	46.16 ± 25.89	3.121	0.002
T3	33.26 ± 17.59	46.23 ± 26.46	2.686	0.009
T5	31.44 ± 15.60	46.48 ± 25.33	3.324	0.001

### Comparison of SAS scores between the two groups

The SAS scores at T0, T1, T2, T3, T4, and T5 of the intervention group were significantly lower than those of the control group (*P* < 0.001; Table 5).

**Table 5:** Comparison of SAS anxiety scores of the families during the perioperative period

Items	Control group(*N* = 43)	Experimental group(*N* = 44)	t	*P*
T0	51.34 ± 13.59	39.83 ± 12.85	-4.058	<0.001
T1	55.64 ± 1.55	40.17 ± 11.68	-5.254	<0.001
T2	50.61 ± 13.29	39.89 ± 12.00	-3.952	<0.001
T3	40.37 ± 12.83	53.34 ± 15.82	-4.206	<0.001
T4	51.72 ± 12.69	38.58 ± 9.33	-5.509	<0.001
T5	51.37 ± 13.96	36.34 ± 9.56	-5.873	<0.001

### Comparison of satisfaction scores between the two groups

The perioperative satisfaction of the patients’ families in the push group was significantly improved compared to that in the control group (Table 6).

**Table 6:** Comparison of satisfaction scores of the families during the perioperative period

Items	Control group(*N* = 43)	Experimental group(*N* = 44)	t	*P*
Satisfaction scores of the perioperative period	93.67 ± 4.34	97.66 ± 2.50	5.264	<0.001

## Discussion

The results of this study indicate that the anxiety levels of family members in the perioperative period were significantly reduced in the experimental group compared to the control group. Real-time sharing of the surgical process refers to a service that meets the information needs of patients and their families throughout the surgery. The aim is to familiarize patients and their families with the anesthetic surgical environment, expert information, precautions during surgery, knowledge about related diseases, postoperative precautions, health care information, etc., to alleviate patients’ nervousness and fear, guide patients and their families to cooperate and help each other, and ensure patient safety during the perioperative period. Research by Duan Weili et al. [20] has proven that during the novel coronavirus period, through information management, making surgical progress nodes visible can reduce the back and forth running of family members and lower the risk of infection. Baydemir et al. [21] also advocate for using short messages to regularly communicate surgical progress information with patients’ families. The results show that this can alleviate the anxiety levels of patients’ families and increase their satisfaction.

After the release of surgical progress information, the patient’s family members do not have to anxiously wait in the waiting area but can wait quietly in the ward for the information to be released. This study uses online communication to inform family members about the dissemination of surgical knowledge and the simultaneous release of surgical information. The aim is to ensure the accurate release and timely update of the patient’s surgical status information. The interface is simple and clear, making it easy for family members to obtain relevant surgical information. This intervention has been validated for its effects on family members’ anxiety levels, postoperative nursing satisfaction, hospital environment management, and patient recovery. The level of anxiety of the patient’s family members is related to their understanding of the disease and their knowledge of psychotherapy.

Family members of children undergoing general anesthesia have a high level of anxiety during the perioperative period. The process of the child’s surgery is the main cause of family anxiety. The main factors for low patient satisfaction during the perioperative period are service attitude, medical effects, and medical communication. Patients and their families undergo significant psychological changes during the illness, with high expectations. If these are not met, dissatisfaction can easily arise. It is worth noting that in this study, the highest anxiety scores for the family members of the children were when the child entered the operating room. The longer the surgery, the higher the level of anxiety, which is consistent with the research results of Munday [22] and Loghmani [23]. Although accompaniment in the anesthesia recovery room can improve satisfaction to a certain extent, it is not enough to alleviate the anxiety of the family members of the children throughout the perioperative period [24]. If family members can participate in the guidance and psychological care of the children during the peri-anesthesia period, it will have a positive effect on the child’s recovery.

In addition, the experimental group also showed better information satisfaction and participation. Not only the patient but also the person accompanying the patient should be taken care of [25]. The surgical process is often a period of anxiety and tension for patients and their families. However, real-time sharing of the surgical process can help alleviate this anxiety and provide better psychological support. In many medical institutions, real-time sharing of the surgical process can be achieved through the use of the internet and communication technology. This sharing can be done through video streaming or real-time message transmission. The patient’s family members can watch the surgical process in real time through a dedicated application installed on their mobile phones or computer. Family members can obtain timely information about the progress of the surgery. Through real-time sharing, doctors or nurses can regularly send updates to family members, informing them of the progress and status of the surgery. This can alleviate the anxiety of family members, let them know whether the surgery is going smoothly, and provide reassurance and support.

Therefore, using real-time sharing of surgical progress information as a means to reduce the perioperative anxiety of families of elective surgery children is effective and beneficial. This study provides a feasible method for improving the family experience during pediatric surgery.

### Limitations of the study

This study has some limitations. First, due to conditions, it is a single-center study, and multicenter studies should be conducted to make the data more substantial. Second, it mainly assessed the early anxiety level of family members of perioperative children and did not follow up on changes in the medium- and long-term anxiety levels of family members. Third, it did not consider the impact of intraoperative medication and the child’s underlying diseases on the perioperative anxiety level of family members. In addition, this research plan is not a double-blind design, and the staff cannot ignore the patient’s grouping situation and intervention measures (real-time push of surgical progress), and there is a risk of evaluation bias.

Generally, families will wait outside the surgery district, and the movement of persons could increase the possibility of nosocomial infection. If we use a information system to send the status of children, they don’t need to wait outside the surgery district but to stay in the ward. But we didn’t set a variable to assess the improving of the hospital environment. Therefore, the real-time sharing in decreasing the hospital environment is just in theory. And it should be assessed in further study by specifically variables.

Of course, real-time sharing of the surgical process also has some limitations and considerations. First, family members must obtain the patient’s consent to participate in real-time sharing. The protection of the patient’s private information and respect for privacy rights are crucial. Second, medical institutions must ensure that real-time sharing does not interfere with or affect the surgical process.

## Conclusions and practical implications

Real-time sharing of surgical progress can help alleviate the anxiety of families during surgery. This sharing can provide information and support and enhance communication and unity. We hope that the next step of the research will increase the push of surgical progress in adult patient families to control their anxiety.

## Data Availability

The datasets used and/or analyzed during the current study are available from the corresponding author on reasonable request.

## Abbreviations

ASA: American Society of Anesthesiology
PSQI: Pittsburgh Sleep Quality Index
SAS: Self-rating anxiety scale
SOC-13: State of Cohesion-13 Scale
